# Bed rest for preventing complications after transfemoral cardiac catheterisation: a protocol of systematic review and network meta-analysis

**DOI:** 10.1186/s13643-015-0036-0

**Published:** 2015-04-15

**Authors:** Alberto Dal Molin, Fabrizio Faggiano, Fabio Bertoncini, Giulia Buratti, Erica Busca, Roberta Casarotto, Samanta Gaboardi, Elias Allara

**Affiliations:** School of Nursing, Biella Hospital, Corso Pella 10, 13900 Biella, Italy; Department of Translational Medicine, Università del Piemonte Orientale, Via Salaroli 17, 28100 Novara, Italy; Internal Medicine, Ospedale degli Infermi, Via Dei Ponderanesi 2, 13875 Ponderano - Biella, Italy; Nursing Home ‘Villa Poma’, Viale della Rimembranza 7, 13816 Miagliano, Biella Italy; Inpatient Neonatology/Neonatal Intensive Care Unit, Azienda Ospedaliero-Universitaria Città della Salute e della Scienza di Torino, Ospedale Ostetrico Ginecologico S.Anna, Corso Spezia 60, 10126 Torino, Italy; Semi Intensive Medicine and Nephrology, Ospedale degli Infermi, Via Dei Ponderanesi 2, 13875 Ponderano - Biella, Italy; Emergency Room, Ospedale degli Infermi, Via Dei Ponderanesi 2, 13875 Ponderano - Biella, Italy; School of Public Health, University of Torino, Via Santena 5 bis, 10126 Torino, Italy

**Keywords:** Bed rest, Complications, Cardiac catheterisation, Nursing, Comfort

## Abstract

**Background:**

Transfemoral cardiac catheterisation is an invasive medical procedure used for therapeutic or diagnostic purposes. Postoperative bed rest can prevent a number of complications such as bleeding and haematoma formation and can result in side effects such as back pain and urinary discomfort. Currently, there is no consensus regarding the optimal length of bed rest. Our objective is to assess the effects of post-catheterisation length of bed rest on bleeding and haematoma, other vascular complications, patient symptoms and patient discomfort, among patients who underwent transfemoral cardiac catheterisation.

**Methods:**

We wrote this protocol in accordance with the Preferred Reporting Items for Systematic Reviews and Meta-Analyses Protocols statement. We defined the search query by using the PICO framework (Population: Patients undergoing cardiac catheterisation; Intervention: early mobilisation; Comparison: late mobilisation; Outcomes: early and late complications). We will search six biomedical databases and five online registries to obtain both published and unpublished studies. We will include randomised controlled trials and quasi-randomised controlled trials, and their quality will be independently appraised with the Cochrane Effective Practice and Organisation of Care criteria for quality assessment. We will carry out a pairwise meta-analysis and network meta-analysis to estimate the overall intervention effects from both direct and indirect comparisons.

**Discussion:**

This review may have considerable implications for practice and help to achieve an effective and efficient management of patients who underwent cardiac catheterisation. This review will be grounded in an expanded search of 11 resources and will employ innovative statistical methods such as network meta-analysis.

**Systematic review registration:**

PROSPERO registration number: CRD42014014222.

**Electronic supplementary material:**

The online version of this article (doi:10.1186/s13643-015-0036-0) contains supplementary material, which is available to authorized users.

## Background

Cardiac catheterisation comprises a group of therapeutic or diagnostic procedures in which placement of cardiac catheter is performed by skin puncture rather than by incision [1]. Examples of such procedures include angiography, which is undertaken for diagnostic purposes, and percutaneous coronary intervention, which is carried out for both diagnostic or therapeutic purposes [2]. It is estimated that approximately 2.2 million patients receive percutaneous coronary intervention worldwide every year [3].

In such procedures, transfemoral puncture is the most common approach because of the larger diameter of such artery [2,4-8]. To reduce complications, manual or mechanical application of a firm pressure above the puncture site is needed [9]. Bed rest in recumbent position and immobilisation of the affected leg are also required for such patients after sheath removal [10,11]. Restricted bed rest and leg immobilisation have been considered essential to reduce the risk of developing complications [2].

Currently, length of bed rest differs according to sheath size and local policy or individual experience [2,7,12-14]. Although both minor and major vascular complications should be prevented, prolonged bed rest has been identified as the most difficult component of post-cardiac catheterisation care [15]. The most frequent complaints of patients during prolonged bed rest are back pain and urinary discomfort, including urinary retention and difficulty with evacuation when in recumbent position [2,10,16]. Patient anxiety and anger due to the unmet needs for comfort are also often noted [17,18].

### Objectives

This study aims to assess the effects of post-catheterisation length of bed rest on bleeding and haematoma, other vascular complications, patient symptoms and patient discomfort, among patients who underwent transfemoral cardiac catheterisation.

## Methods/design

We have written this protocol in accordance with the Preferred Reporting Items for Systematic Reviews and Meta-Analyses Protocols (PRISMA-P) [19], and we will conduct this systematic review in accordance with the Preferred Reporting Items for Systematic Reviews and Meta-Analyses (PRISMA) [20] statement. We will include both randomised controlled trials and quasi-randomised controlled trials with at least two study groups. We have defined the search query by using the following PICO framework [21] (see Table 1).

**Table 1:** **PICO**

**PICO framework**	**Description**
Population	Patients undergoing transfemoral cardiac catheterisation. We will not apply restrictions with regard to patient age, gender, race, comorbidity, setting or other characteristics.
Interventions and comparison	Lengths of bed rest. Since definition of ‘early’ or ‘late’ mobilisation may differ across studies, we will define intervention and comparison groups on the basis of six categories observed in a previous work [22]: 0 to 1.9 h, 2 to 3.9 h, 4 to 5.9 h, 6 to 7.9 h, 8 to 11.9 h, 12 h or greater.
Outcomes	Common and early complications such as bleeding, haematomas. Late complications such as pseudoaneurysms and arteriovenous fistulae. Patient symptoms such as back pain, urinary discomfort and patient discomfort.

### Participants

The study population will include patients undergoing transfemoral cardiac catheterisation. There will be no restrictions by patient age, gender, race, comorbidity, healthcare setting or other characteristics.

### Interventions and comparisons

The intervention of interest is duration of bed rest, defined as lying in bed without permission to get up for any reasons. We will compare early mobilisation with late mobilisation which will be defined based on the studies included in a previous work [22]: 0 to 1.9 h, 2 to 3.9 h, 4 to 5.9 h, 6 to 7.9 h, 8 to 11.9 h, 12 h or greater. We anticipate that for some studies published after the review of Allen et al. [22], bed rest duration might fall into the same category for two or more study arms of the same study, preventing estimation of intervention effect. In such a case, we would split categories to allow the greatest number of studies to be included, while keeping the number of categories as low as possible^a^. This approach will (i) allow a more objective classification of intervention categories, as definitions of ‘early mobilisation’ and ‘late mobilisation’ may vary across studies; (ii) allow inclusion of the studies whose both arms fall into one of the categories observed in Allen et al. [22]; and (iii) favour reasonable compatibility with previous work.

### Outcome

In accordance with Saldanha et al. [23], we define outcomes by five items: domain, specific measurements, specific metric, method, time point.

#### Primary outcomes

*Domain*: bleeding.

*Specific measurement*: number of patients presenting visible areas of bleeding, oozing or haemorrhage at the puncture site.

*Specific metric*: value at time point.

*Method of aggregation*: percent.

*Time point*: 24 h after transfemoral cardiac catheterisation, or closest time point.

*Domain*: haematomas.

*Specific measurement:* number of patients presenting visible ecchymosis or haematomas at the puncture site, or ultrasound-confirmed palpable haematomas at the puncture site.

*Specific metric:* value at time point.

*Method of aggregation*: percent.

*Time point*: 24 h after transfemoral cardiac catheterisation, or closest time point.

#### Secondary outcomes

*Domain*: pseudoaneurysm.

*Specific measurement*: number of patients presenting pseudoaneurysm at the puncture site that can be confirmed by ultrasound.

*Specific metric*: value at time point.

*Method of aggregation*: percent.

*Time point*: 2 weeks after transfemoral cardiac catheterisation, or closest time point.

*Domain*: arteriovenous fistulae.

*Specific measurement*: number of patients presenting arteriovenous fistulae at the puncture site that can be confirmed by ultrasound.

*Specific metric*: value at time point.

*Method of aggregation*: percent.

*Time point*: 2 weeks after transfemoral cardiac catheterisation, or closest time point.

*Domain*: back pain.

*Specific measurement*: Numerical Rating Scale or other scale described by each trial.

*Specific metric*: value at time point.

*Method of aggregation*: mean and standard deviation.

*Time point*: 24 h after transfemoral cardiac catheterisation, or closest time point.

*Domain*: urinary discomfort.

*Specific measurement*: subjective scales of urinary discomfort described by each trial.

*Specific metric*: value at time point.

*Method of aggregation*: mean and standard deviation.

*Time point*: 24 h after transfemoral cardiac catheterisation, or closest time point.

*Domain*: patient discomfort.

*Specific measurement*: subjective scales of patient discomfort described by each trial.

*Specific metric*: value at time point.

*Method of aggregation*: mean and standard deviation.

*Time point*: 24 h after transfemoral cardiac catheterisation, or closest time point.

### Search methods for identification of studies

#### Electronic searches

We will search six biomedical and nursing research databases: MEDLINE, EMBASE, Cochrane Database of Systematic Reviews, CINAHL, SCOPUS, SciELO. Search strategies are shown in Additional file 1.

#### Searching other resources

We will explore five registries of studies to obtain both published and unpublished works (grey literature): National Health Service (NHS) evidence, http://www.uptodate.com, http://clinicaltrials.gov/, http://www.who.int/ictrp/en/, http://www.controlled-trials.com/.

We will manually inspect previous reviews to obtain relevant works from their list of references.

We will populate search results including authors, title and abstracts into Thomson Reuter’s EndNote software, and any duplicates will be automatically removed. Two authors will independently screen titles and abstracts, and any disagreement will be solved by a third author. We will not set any language restrictions and will carry out translations whenever necessary.

We will retrieve the full texts of selected articles, and two authors will independently evaluate whether they are pertinent to this review. Any disagreement will be solved by a third author.

We will assess agreement between the two screeners with Cohen’s kappa coefficient. Agreement will be considered poor if K is lower than 0.20; fair if between 0.21 and 0.40; moderate if between 0.41 and 0.60; good if between 0.61 and 0.80; and very good if equal to or greater than 0.81 [24].

### Quality assessment of primary studies

We will use the Cochrane Effective Practice and Organisation of Care (EPOC) Risk of Bias tool [25], which is a two-part tool addressing specific domains such as: sequence generation and allocation concealment (selection bias), blinding of outcome assessor (detection bias), selective outcome reporting (reporting bias) and other sources of bias. The first part of the tool is intended to describe what was reported to have happened in the study. The second part of the tool involves assigning a judgement related to the risk of bias for each entry, in terms of low, high or unclear risk. See Additional file 2 for details.

### Data extraction

Once articles have been selected for inclusion, data extraction will take place. Two authors will independently extract data with a standard Excel extraction form in which all relevant data will be included for each study. Any disagreement in this phase will be solved by discussion with a third author.

Data collection will comprise four main areas of information:

1. Article:
  * Title
  * Author
  * Year of publication
  * Journal
2. Study characteristics:
  * Setting and location of the study
  * Number of patients
  * Mean patient age
  * Duration of bed rest
  * Purposes of procedure: diagnostic or therapeutic intervention
  * Setting: elective or emergency
  * Size of catheter (potential effect modifier)
  * Presence of procedures to promote haemostasis (potential effect modifier)
  * Study design (randomised or quasi-randomised)
3. Results (divided by duration of bed rest):
  * Number of patients per study group
  * Number of patients presenting active bleeding, including oozing and haemorrhage
  * Number of patients presenting haematomas or ecchymosis
  * Number of patients presenting late vascular complications such as pseudoaneurysms and arteriovenous fistulae
  * Mean and standard deviation of patient discomfort scales
  * Mean and standard deviation of urinary discomfort scales
  * Mean and standard deviation of back pain scales
4. Notes:
  * Language of the study and any other information relevant to this review.

### Measures of treatment effect

We will analyse dichotomous outcomes by calculating the risk ratio (RR) for each included trial, along with the corresponding precision of effect estimate expressed by their 95% confidence intervals. For continuous outcome measures, we expect to pool studies which use different scales and will thus compute standardised mean difference (SMD) and its corresponding 95% confidence interval for each included trial.

### Dealing with missing data

Wherever possible, we will contact the authors of the studies for integration of any missing data. If we are unable to obtain information regarding at least one of the primary outcomes, we will include the study from the review and exclude it from the meta-analysis. If we are unable to obtain information needed for quality assessment, we will consider the EPOC criterion of interest to be at a high or unclear risk of bias, depending on the information already available for that study.

### Assessment of reporting biases

If the number of studies will be sufficient, funnel plots (that is, scatter plots of the effect estimate from each study against the standard error) will be used to assess the potential for bias related to the size of the trials, which could indicate possible publication bias.

### Data synthesis

#### Pairwise meta-analysis

A traditional frequentist pairwise meta-analysis will be carried out by pooling parameter estimates of included studies. Due to the large variability in clinical approaches for cardiac catheterisation, we will use a random effects model to account for potential between-study heterogeneity. We will perform frequentist pairwise meta-analysis in R using the metafor package [26].

#### Network meta-analysis

We anticipate that a network meta-analysis will be performed to allow estimation of the relative effects of every duration of bed rest regardless of whether they have been compared directly in head-to-head trials.

##### General approach

We will perform frequentist network meta-analysis in R using the mvmeta package [27] for most analyses. For analyses not yet feasible with the above-mentioned package, we will use the mvmeta command [28] in STATA 13, including self-programmed STATA routines available at http://www.mtm.uoi.gr.

##### Assessment of statistical heterogeneity

We will assume a common estimate for the heterogeneity variance across comparisons. The assessment of statistical heterogeneity in the entire network will be based on the magnitude of the heterogeneity variance parameter (τ^2^) estimated from the network meta-analysis models. We will also estimate a total I-squared value for heterogeneity in the network [29].

##### Transitivity

To infer about the assumption of transitivity, we will assess whether the included bed rest durations are similar when they are evaluated in RCTs with different designs, and we will compare the distribution of the potential effect modifiers (for example, size of catheter and presence of procedures to promote haemostasis) across the different pairwise comparisons [30].

##### Assessment of local statistical inconsistency

We will use a loop-specific approach to evaluate the consistency assumption in each closed loop of the network. In our study, a loop of evidence would be formed by at least three categories of bed rest duration which have been compared in studies. A loop-specific approach evaluates the consistency assumption in each closed loop separately as the difference between direct and indirect estimates for a specific comparison in the loop (inconsistency factor). We will look at the 95% confidence interval of the inconsistency factor to infer whether there is evidence of a difference between direct and indirect estimates. We will assume a common heterogeneity estimate within each loop [28,31]. We will interpret findings with caution due the increased likelihood of type I error arising from multiple testing [32].

##### Assessment of global statistical inconsistency

To check the assumption of consistency in the entire network, we will use a design-by-treatment model [33]. This method accounts for different sources of inconsistency that can occur when studies with different designs (for example, two-arm trials *vs*. three-arm trials) give different results as well as disagreement between direct and indirect evidence. Using this approach, we will infer about the presence of inconsistency from any source in the entire network based on a chi-square test. To distinguish between inconsistency and heterogeneity, we will employ the I-squared for inconsistency that measures the percentage of variability that cannot be attributed to random error or heterogeneity (within comparison variability).

##### Dealing with heterogeneity and inconsistency

If we find important heterogeneity or/and inconsistency, we will explore the possible sources. If sufficient studies are available, we will perform meta-regression by including potential effect modifiers of the associations between bed rest duration and outcome [30].

### Sensitivity analysis

To incorporate the assessment of risk of bias in the review process, we will plot intervention effects estimates for different outcomes stratified for risk. If we find differences in results by risk of bias and if the number of included studies is sufficient, we will perform sensitivity analysis excluding studies with high risk of bias from the analysis.

## Discussion

The aim of this systematic review is to assess the effects of different lengths in bed rest after cardiac catheterisation on patient-level outcomes such as vascular complications, symptoms and satisfaction. Knowing to what extent different lengths of bed rest can affect quality of care and patient comfort may have considerable implications for practice, by grounding clinical decision in a transparently selected pool of studies and by allowing more efficient, evidence-based management of resources for millions of patients worldwide [1].

We feel that publication of our review protocol prior to conduction of our systematic review would encourage transparency and would reduce risk of selection bias, as well as reducing the possibility of methodological inaccuracies.

Our work would also have the advantage of considering for inclusion studies indexed in the EMBASE dataset, a large biomedical database which was not searched in a previous review [34,35]. Since we plan to consider for inclusion also studies embed in previous reviews, our review will be built on top of previous work, likely expanding and updating their evidence base.

Lastly, it seems reasonable to expect that some studies might report the effects of different duration of bed rest, with considerable heterogeneity for both the intervention group and the comparison group. In such a case, our review would summarise data by using a network meta-analysis approach. This methodology would allow obtaining effect estimates from both direct and indirect comparisons, thus allowing more accurate predictions which are likely to result in more reliable recommendations about the optimal length of bed rest after cardiac catheterisation.

## Endnote

^a^For example, if a study compared 8 h*vs*. 10 h, both arms would fall into the same category (‘8 to 11.9 h’) defined according to a previous study [22]. We would thus split that category into two new categories (for example, ‘8 to 9.9 h’ and ‘10 to 11.9 h’), to allow comparison of study arms. If another study compared 8-h bed rest duration with 9.5 h, then both study arms would fall into the ‘8 to 9.9 h’ category. We would therefore change the ‘8 to 9.9 h’ category into ‘8 to 9.4 h’ and the ‘10 to 11.9 h’ category into ‘9.5 to 11.9 h’, in order to keep the number of categories of bed rest duration as small as possible while allowing inclusion of as many studies possible.

## Additional files

Additional file 1:
**Search strategies.**
Additional file 2:
**EPOC criteria for quality assessment studies with a separate control group (RCTs, CCTs, CBAs).**

## Abbreviations

CBAs: Controlled before and after studies
CCTs: Controlled clinical trials
EPOC: Cochrane effective practice and organisation of care
NHS: National health service
OR: Odds ratio
PICO: Population, intervention, comparison, outcome
PRISMA: Preferred reporting items for systematic reviews and meta-analyses
RCTs: Randomised control trials
SMD: Standardised mean difference
