# Supplementary material for: Bed rest for preventing complications after transfemoral cardiac catheterisation: a protocol of systematic review and network meta-analysis
Source: Syst Rev. 2015 Apr 15;4:47. doi: 10.1186/s13643-015-0036-0 (PMC4406333; doi:10.1186/s13643-015-0036-0)
Supplement: Additional file 2: — EPOC criteria for quality assessment studies with a separate control group (RCTs, CCTs, CBAs). [file 13643_2015_36_MOESM2_ESM.doc]

Additional file 2. EPOC Criteria for Quality Assessment studies with a separate control group (RCTs, CCTs, CBAs)

Further information can be obtained from the Cochrane handbook section on risk of bias [25].

| Criterion | Score | | |
| --- | --- | --- | --- |
|  | Low risk | High risk | Unclear risk |
| Random sequence generation (selection bias) | If a random component in the sequence generation process is described (for example, Referring to a random number table). | When a non-random method is used (for example, performed by date of admission). CCTs and CBAs should be scored ‘No.’ | If not specified in the paper. |
| Allocation concealment (selection bias) | If the unit of allocation was by institution, team or professional and allocation were performed on all units at the start of the study; or if the unit of allocation was by patient or episode of care and there was some form of centralised randomisation scheme, an on-site computer system or sealed opaque envelopes were used. | CBAs should be scored ‘No.’ | If not specified in the paper. |
| Blinding of outcome assessor (detection bias) | If the authors state explicitly that the primary outcome variables were assessed blindly, or the outcomes are objective, for example, length of hospital stay. Primary outcomes are those variables that correspond to the primary hypothesis or question as defined by the authors. | If the outcomes were not assessed blindly. | If not specified in the paper. |
| Selective reporting (reporting bias) | If there is no evidence that outcomes were selectively reported (for example, all relevant outcomes in the ‘Methods’ section are reported in the ‘Results’ section). | If some important outcomes are subsequently omitted from the results. | If there is insufficient information to permit judgement of ‘low risk’ or ‘high risk.’ |
| Other risks of bias | If there is no evidence of other risk of biases | ¬ | ¬ |
